# Lobectomy with ipsilateral central lymph node dissection might be an appropriate surgical method for select cases of isthmic papillary thyroid carcinoma: a retrospective study with propensity scores matching analysis

**DOI:** 10.3389/fendo.2025.1588323

**Published:** 2025-07-10

**Authors:** Bin Wang, Chun-Rong Zhu, Hong Wen, Yuan Fei, Zhu-Juan Wu, Hong Liu, Xin-Min Yao, Jian Wu

**Affiliations:** ^1^ Center of Breast and Thyroid Surgery, Department of General Surgery, The Third People’s Hospital of Chengdu, Chengdu, Sichuan, China; ^2^ Department of Oncology Ward 2, The Third People’s Hospital of Chengdu, Chengdu, Sichuan, China; ^3^ Department of Ultrasound, The Third People’s Hospital of Chengdu, Chengdu, Sichuan, China

**Keywords:** isthmus, papillary thyroid carcinoma, contralateral paratracheal lymph node metastasis, surgical method, propensity score matching

## Abstract

**Objective:**

The study aimed to ascertain the appropriate surgical method for isthmic papillary thyroid carcinoma (PTC).

**Methods:**

We reviewed the records of patients who underwent thyroid surgery for PTC in our institution from July 2018 to June 2024. The isthmus was categorized into central isthmus and paracentral isthmus. Data were compared to explore the risk factors of contralateral paratracheal lymph node metastasis (LNM) and the presence of more than 5 metastatic lymph nodes between patients with paracentral isthmic PTC and those with lobar PTC, and between patients with paracentral isthmic PTC and those with central isthmic PTC. Propensity score matching was used to identify a cohort of patients with similar baseline characteristics among patients with paracentral isthmic PTC and lobar PTC to minimize discrepancies in the number between the two groups.

**Results:**

Prelaryngeal and/or pretracheal LNM was confirmed to be an independent risk factor for contralateral paratracheal LNM (OR = 3.43; 95%CI 1.74 – 8.92; p = 0.013) and presence of more than 5 metastatic lymph nodes (OR = 4.55; 95%CI 1.46 – 14.15; p = 0.009) in patients with paracentral isthmic PTC and lobar PTC. While, the location in the paracentral isthmus did not exhibit a significant association with them in these patients. Conversely, being located in the central isthmus was confirmed to be a risk factor for contralateral paratracheal LNM (OR = 4.67; 95%CI 1.53 – 14.21; p = 0.007) and the presence of more than5 metastatic lymph nodes (OR = 4.55; 95%CI 1.46 – 14.15; p = 0.009) among patients with isthmic (central and paracentral) PTC.

**Conclusion:**

Lobectomy with ipsilateral central lymph node dissection might be appropriate for paracentral isthmic PTC without prelaryngeal and pretracheal LNM. Total thyroidectomy with bilateral central lymph node dissection might be necessary for central isthmic PTC.

## Introduction

Thyroid gland consists of bilateral lobes and isthmus and is H-shaped. Thyroid carcinoma might occur in anywhere the gland. While the surgical method was mainly based on the carcinoma size in the 2015 American Thyroid Association Management Guidelines for Adult Patients with Thyroid Nodules and Differentiated Thyroid Cancer, which could meet the guidance for lobar papillary thyroid carcinoma (PTC) (1). For isthmic PTC, there were no surgical methods to match (1).

Some research suggested that isthmic PTC was more aggressive compared to lobar PTC (2–9). While some other studies suggested that being located in isthmus was not associated with the invasiveness of PTC and less-than-total thyroidectomy might be adequate for isthmic PTC (10–16). The surgical scope of isthmic PTC remains controversial.

Here, we reported a retrospective study where data were compared between different locational PTC. The study aimed to find the appropriate surgical method for isthmic PTC by exploring the risk factors of lymph node metastasis (LNM) for isthmic PTC.

## Patients and methods

### Patients

We reviewed the records of patients who underwent thyroid surgery for PTC in our institution from July 2018 to June 2024. Patients who met all the following criteria were included: 1) those who underwent initial thyroid surgery; 2) those with isthmic PTC or unilateral lobar PTC; 3) the size of PTC ≤ 4 cm; 4) those with negative lateral lymph node according to the preoperative imageological examination. The following patients were excluded: 1) those with a history of other neck surgery; 2) those with a history of neck radiation therapy; 3) those with concurrent parathyroid diseases or abnormal preoperative serum parathyroid hormone (PTH) levels; 4) those with incomplete medical and follow-up records in postoperative 6 months. Approval for this study was obtained from the Medical Ethics Committee of The Third People’s Hospital of Chengdu, with informed consent routinely acquired from all participants for utilizing clinical data in medical research upon discharge.

### Surgical strategies and procedures

According to the Chinese Guidelines for the Diagnosis and Management of Thyroid Nodules and Differentiated Thyroid Cancer (Second edition) (17), ipsilateral central neck dissection (ICND) is standard procedure for all cases of PTC in our hospital. The surgical strategies were delineated as follows. For unilateral cT1~2N0 PTC, the surgical strategies were detailed in a prior study (18). For isthmic PTC, total thyroidectomy (TT) with bilateral central lymph node dissection (BCND) was performed. For T3b~4 PTC and cN1a PTC, TT with ICND was routinely performed, and contralateral central neck dissection was performed upon confirmation of extraglandular invasion and/or prelaryngeal and/or pretracheal LNM through intraoperative frozen pathology. The surgical procedures for thyroidectomy and central lymph node dissection adhered to the description outlined in previous literature (18).

### Perioperative management and data collection

**Figure 1:**
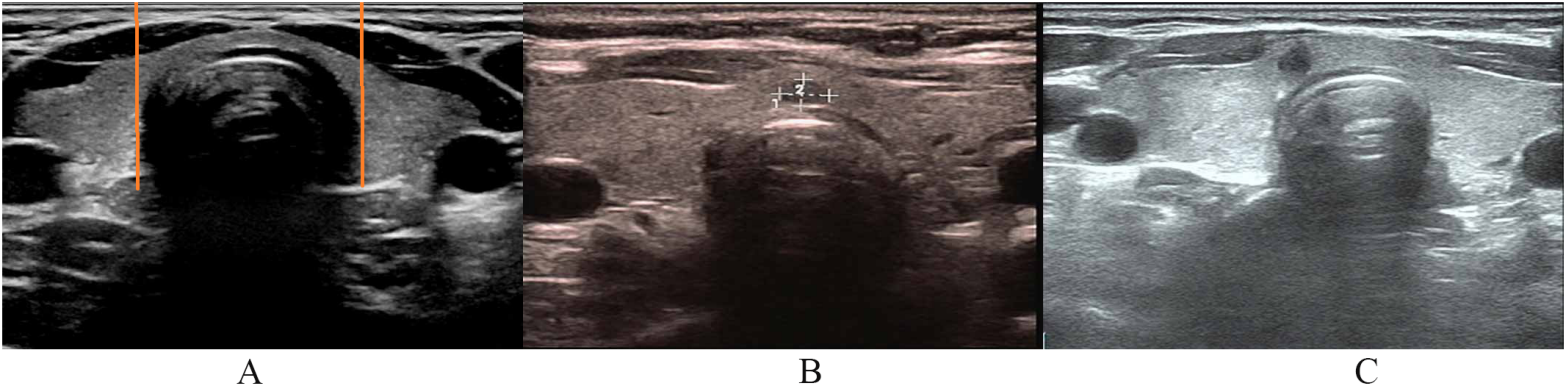
Thyroid isthmus and isthmic papillary thyroid carcinoma. **(A)** The definition of thyroid isthmus. **(B)** Central isthmic papillary thyroid carcinoma. **(C)** Paracentral isthmic papillary thyroid carcinoma.

It was applied that standardized perioperative management and postoperative calcium supplementation strategies to all patients as outlined in a previous study (19). Thyroid isthmus is defined as the gland between the two vertical virtual lines from the lateral border of the trachea to the skin surface in the ultrasound cross-sectional image (**Figure 1**). Central isthmus is defined as the middle 1/3 of the isthmus, and the other is regarded as the paracentral isthmus. The location of PTC depended on the center of the tumor. A professional ultrasound physician (Hong Wen) reviewed the preoperative ultrasound images and made the classification of paracentral and central isthmic regions. Hypoparathyroidism was considered when serum PTH levels fell below the normal range (1.6–6.9 pmol/L) or necessitated calcium supplementation due to symptomatic presentations, irrespective of PTH values. Immediate hypoparathyroidism was defined as onset within 24 hours post-surgery, with durations exceeding 1 and 6 months classified as protracted and permanent hypoparathyroidism, respectively. Data acquisition encompassed demographic data, comorbidities, preoperative evaluations, surgical details, number of autoplastic and/or inadvertently resected parathyroid gland (PG), postoperative pathology reports, lesion characteristics, postoperative complications, and postoperative PTH levels.

### Statistical analysis

All analyses were conducted using SPSS version 26.0 software (SPSS Inc, Chicago, IL, USA). Continuous data and categorical data were expressed as mean ± standard deviation (SD) and absolute numbers, respectively. Continuous variables were analyzed using Student t-tests or Mann–Whitney U tests, while categorical variables were evaluated using Pearson chi-square tests or Fisher exact tests. Multivariate binary logistic regression analysis was employed to identify risk factors for contralateral paratracheal LNM and the presence of more than 5 metastatic lymph nodes. Statistical significance was defined as P < 0.05.

Due to the wide difference in the number between paracentral isthmic PTC and lobar PTC, propensity score matching (PSM) was used to identify a cohort of patients with similar baseline characteristics. PSM was achieved by using Stata version 14.0 (Stata Corp LP, College Station, TX, USA). Matching was performed with the use of a 1:3 matching protocol to minimize the selection bias and confounding differences, with the caliper value set to 0.02. The following variables were included to match: gender, age, body mass index, hypertension, diabetes, hypothyroidism, hyperthyroidism, Hashimoto’s thyroiditis, nodular goiter, left or right, the largest size of the tumor, preoperative lymphadenectasis in the central, the largest size of the intumescent lymph node in the central zone.

## Results

A total of 816 consecutive patients were included in this study. Among them the lesion located in the lobe in 732, paracentral isthmus in 59, central isthmus in 25. Patients with isthmic PTC underwent TT with BCND. Among patients with lobar PTC, 90 patients underwent lobe thyroidectomy with ICND, 299 patients TT with ICND, and 342 patients TT with BCND. With the use of PSM, 173 patients with lobar PTC were matched with 59 patients with paracentral isthmic PTC. Before matching, the mean propensity score was 0.025 for patients with lobar PTC and 0.035 for patients with paracentral isthmic PTC. Then the mean propensity scores were both 0.035 after matching.

Comparisons were made between lobar PTC and paracentral isthmic PTC patient cohorts (**Table 1**). Before PSM, the paracentral isthmic PTC group showed higher incidences of PG autotransplantation (72.9% vs 57.4%, P = 0.02; **Table 1**), inadvertent PG resection (20.3% vs 10.0%, P = 0.013; **Table 1**), postoperative immediate hypoparathyroidism (66.1% vs 30.3%, P < 0.001; **Table 1**), prelaryngeal and/or pretracheal LNM (42.4% vs 29.5%, P = 0.039; **Table 1**), and contralateral paratracheal LNM (18.6% vs 9.2%, P = 0.019; **Table 1**). And the average number of metastatic lymph nodes was also higher in the paracentral isthmic PTC cohort than in the lobar PTC group (3.6 ± 2.8 vs 2 ± 2.6, P = 0.045; **Table 1**). After PSM, only the incidences of inadvertent PG resection and postoperative immediate hypoparathyroidism were higher in the paracentral isthmic PTC cohort than the lobar PTC group (20.3% vs 9.8%, P = 0.035; 66.1% vs 39.9%, P < 0.001; **Table 1**). Incidences of prelaryngeal and/or pretracheal LNM, contralateral paratracheal LNM, and the presence of more than5 metastatic lymph nodes did not significantly vary between the groups.

**Table 1:** he characteristics of the group of lobar PTC and paracentral isthmic PTC (before and after matching).

Variables	Before matching			After matching		
	Lobar PTC (n = 732)	Paracentral isthmic PTC (n = 59)	P	Lobar PTC (n = 173)	Paracentral isthmic PTC (n=59)	P
Gender (M/F)	206/526	22/37	0.136	52/121	22/37	0.303
Age (years old)	43.8 ± 12.5	42.8 ± 12.5	0.738	45.4 ± 13.7	42.8 ± 12.5	0.501
≥55y	139	11	0.948	34	11	0.866
<55y	593	48		139	48	
BMI	23.4 ± 3.4	22.1 ± 3.2	0.129	22.3 ± 2.6	22.1 ± 3.2	0.758
Hypertension	75	5	0.664	16	5	0.858
Diabetes	19	2	0.666	7	2	>0.99
Hyperthyroidism	11	0	0.711	0	0	—
Hypothyroidism	23	1	0.819	2	1	>0.99
Hashimoto’s Thyroiditis	209	18	0.749	48	18	0.685
Nodular goiter	386	28	0.435	87	28	0.707
Left lobe/Right lobe	408	29	0.328	93	29	0.541
Largest size of the main lesion (mm)	11.9 ± 7.2	14.5 ± 8.3	0.159	14.5 ± 7.6	14.5 ± 8.3	0.992
>10mm	327	30	0.359	82	30	0.647
≤10mm	405	29		91	29	
Extraglandular invasion	15	3	0.294	4	3	0.526
Preoperative lymphadenectasis in the central zone	101	12	0.167	30	12	0.606
Largest size of lymph nodes (mm)	11.3 ± 5.1	11 ± 5.7	0.933	11.1 ± 5.3	11 ± 5.7	0.966
Transient hoarseness	22	0	0.348	2	0	>0.99
Permanent hoarseness	4	0	>0.99	0	0	—
Autoplastic PG	0.7 ± 0.7	1.1 ± 0.8	0.016	0.8 ± 0.7	1.1 ± 0.8	0.072
≥1	420	43	0.02	108	43	0.146
0	312	16		65	16	
Inadvertently removed PG	0.1 ± 0.3	0.2 ± 0.3	0.558	0.1 ± 0.3	0.2 ± 0.3	0.778
≥1	73	12	0.013	17	12	0.035
0	659	47		156	47	
Postoperative PTH						
1 Day	2.33 ± 1.39	1.99 ± 1.24	0.327	2.1 ± 1.44	1.99 ± 1.24	0.773
1 Month	3.85 ± 1.66	4.02 ± 1.48	0.699	3.69 ± 1.67	4.02 ± 1.48	0.51
6 Months	4.16 ± 1.77	3.14 ± 1.25	0.322	3.88 ± 2.15	3.14 ± 1.25	0.577
Immediate hypoparathyroidism	222	39	<0.001	69	39	<0.001
Protracted hypoparathyroidism	29	5	0.19	7	5	0.324
Permanent hypoparathyroidism	8	1	0.504	1	1	0.445
Prelaryngeal and pretracheal LNM	0.6 ± 1.3	1.2 ± 1.1	0.065	0.9 ± 1.1	1.2 ± 1.1	0.326
≥1	216	25	0.039	64	25	0.463
0	516	34		109	34	
Contralateral paratracheal LNM	0.2 ± 0.8	0.6 ± 1.3	0.235	0.2 ± 0.6	0.6 ± 1.3	0.199
≥1	67	11	0.019	17	11	0.073
0	665	48		156	48	
Total number of metastatic lymph nodes	2 ± 2.6	3.6 ± 2.8	0.045	3.0 ± 2.7	3.6 ± 2.8	0.253
>5	79	12	0.27	20	12	0.091
≤5	653	47		153	47	

PTC, papillary thyroid carcinoma; M, male; F, female; BMI, body mass index; PG, parathyroid gland; PTH, parathyroid hormone; LNM, lymph node metastasis.

Analyses of risk factors for contralateral paratracheal LNM and the presence of more than 5 metastatic lymph nodes among patients with lobar PTC and paracentral isthmic PTC were delineated in **Table 2**. Multivariate analysis highlighted young age (≥ 55y; OR = 0.31, 95%CI 0.14 – 0.71; p = 0.005; **Table 2**), larger size (> 1cm) of the largest diameter of tumor (OR = 4.15, 95%CI 2.48 – 6.95; p < 0.001; **Table 2**), extraglandular invasion (OR = 1.46, 95%CI 1.08 – 1.87; p = 0.044; **Table 2**), and prelaryngeal and/or pretracheal LNM (OR = 3.43; 95%CI 1.74 – 8.92; p = 0.013; **Table 2**) as independent risk factors for contralateral paratracheal LNM. And this analysis also indicated that young age (≥ 55y; OR = 0.35, 95%CI 0.16 – 0.76; p = 0.008; **Table 2**), larger size (> 1cm) of the largest diameter of tumor (OR = 5.71, 95%CI 3.29 – 9.92; p < 0.001; **Table 2**), prelaryngeal and/or pretracheal LNM (OR = 4.55; 95%CI 1.46 – 14.15; p = 0.009; **Table 2**), and contralateral paratracheal LNM (OR = 1.63; 95%CI 1.03 – 2.67; p = 0.042; **Table 2**) independently raised the risk of the presence of more than 5 metastatic lymph nodes.

**Table 2:** Multivariate analysis of risk factors for contralateral paratracheal LNM and more than 5 metastatic lymph nodes from data of the group of lobar PTC and paracentral isthmic PTC after matching.

Variables	Contralateral paratracheal LNM (G=479.587, P<0.001)			More than 5 metastatic lymph nodes (G=465.528, P<0.001)		
	OR	95%CI	P	OR	95%CI	P
Advanced age (≥55y)	0.47	0.14 — 0.71	0.005	0.35	0.16 — 0.76	0.008
Larger size of the largest size of the main lesion (>10mm)	5.23	2.48 — 6.95	0.000	5.71	3.29 — 9.92	0.000
Extraglandular invasion	1.43	1.08 — 1.87	0.044			
Prelaryngeal and/or pretracheal LNM	4.67	1.74 — 8.92	0.013	4.55	1.46 — 14.15	0.009
Contralateral paratracheal LNM				1.63	1.03 — 2.67	0.042

LNM, lymph node metastasis; PTC, papillary thyroid carcinoma.

**Table 3** displayed data for patients with paracentral isthmic PTC and central isthmic PTC. The incidence of contralateral paratracheal LNM and the presence of more than 5 metastatic lymph nodes were lower in the paracentral isthmic PTC cohort compared to cases exhibiting bilateral paratracheal LNM and the presence of more than 5 metastatic lymph nodes in the central isthmic PTC group (18.6% vs 40%, P = 0.039; 20.3% vs 44%, P = 0.026; **Table 3**), respectively. Subsequent multivariate analyses indicated that young age (≥ 55y; OR = 0.47, 95%CI 0.23 – 0.78; p = 0.015; **Table 4**), larger size (> 1cm) of the largest diameter of tumor (OR = 5.23, 95%CI 1.93 – 11.35; p = 0.037; **Table 4**), preoperative lymphadenectasis in the central zone (OR = 1.43; 95%CI 1.13 – 2.02; p = 0.007; **Table 4**), and being located in central isthmus (OR = 4.67; 95%CI 1.53 – 14.21; p = 0.007; **Table 4**) were independent risk factors for contralateral paratracheal LNM, and that young age (≥ 55y; OR = 0.54, 95%CI 0.28 – 0.99; p = 0.046; **Table 4**), larger size of the largest diameter of tumor (> 1cm; OR = 7.25, 95%CI 2.33 – 18.57; p = 0.003; **Table 4**), being located in central isthmus (OR = 4.55; 95%CI 1.46 – 14.15; p = 0.009; **Table 4**), prelaryngeal and/or pretracheal LNM (OR = 7.39; 95%CI 2.84 – 23.68; p = 0.009; **Table 4**), and contralateral paratracheal LNM (OR = 3.14; 95%CI 2.46 – 8.01; p = 0.006; **Table 4**) were independent risk factors for presence of more than 5 metastatic lymph nodes.

**Table 3:** The characteristics of the group of paracentral isthmic PTC and central isthmic PTC.

Variables	Paracentral isthmic PTC (n = 59)	Central isthmic PTC (n = 25)	P
Gender (M/F)	22/37	5/20	0.121
Age (years old)	42.8 ± 12.5	42.9 ± 13.5	0.97
≥55y	11	5	>0.99
<55y	48	20	
BMI	22.1 ± 3.2	24.1 ± 3.5	0.051
Hypertension	5	5	0.261
Diabetes	2	1	>0.99
Hyperthyroidism	0	0	—
Hypothyroidism	1	0	>0.99
Hashimoto’s Thyroiditis	18	6	0.546
Nodular goiter	28	17	0.084
Left lobe/Right lobe	29	—	—
Largest size of the main lesion (mm)	14.5 ± 8.3	14.7 ± 5.8	0.919
>10mm	30	13	0.923
≤10mm	29	12	
Extraglandular invasion	3	2	0.631
Preoperative lymphadenectasis in the central zone	12	8	0.251
Largest size of lymph nodes (mm)	11 ± 5.7	10.3 ± 3.9	0.839
Transient hoarseness	0	0	—
Permanent hoarseness	0	0	—
Autoplastic PG	1.1 ± 0.8	1.2 ± 0.9	0.753
≥1	43	18	0.651
0	16	7	
Inadvertently removed PG	0.2 ± 0.3	0.3 ± 0.3	0.452
≥1	12	6	0.708
0	47	19	
Postoperative PTH			
1 Day	1.99 ± 1.24	1.63 ± 1.11	0.306
1 Month	4.02 ± 1.48	3.34 ± 1.48	0.155
6 Months	3.14 ± 1.25	2.69 ± 1.09	0.552
Immediate hypoparathyroidism	39	16	0.853
Protracted hypoparathyroidism	5	3	0.69
Permanent hypoparathyroidism	1	0	>0.99
Prelaryngeal and pretracheal LNM	1.2 ± 1.1	1.6 ± 1.5	0.326
≥1	25	16	0.07
0	34	9	
Contralateral/bilateral paratracheal LNM*	11	10	0.039
Total number of metastatic lymph nodes	3.6 ± 2.8	5.4 ± 3.2	0.072
>5	12	11	0.026
≤5	47	14	

PTC, papillary thyroid carcinoma; M, male; F, female; BMI, body mass index; PG, parathyroid gland; PTH, parathyroid hormone; LNM, lymph node metastasis.

*Contralateral paratracheal LNM in the group of Paracentral isthmic PTC, bilateral paratracheal LNM in the group of central isthmic PTC.

**Table 4:** Multivariate analysis of risk factors for contralateral paratracheal LNM and more than 5 metastatic lymph nodes from data of the group of paracentral isthmic PTC and central isthmic PTC.

Variables	Contralateral paratracheal LNM (G=266.582, P<0.001)			More than 5 metastatic lymph nodes (G=289.056, P<0.001)		
	OR	95%CI	P	OR	95%CI	P
Advanced age (≥55y)	0.47	0.23 — 0.78	0.015	0.54	0.28 — 0.99	0.046
Larger size of the largest size of the main lesion (>10mm)	5.23	1.93 — 11.35	0.037	7.25	2.33 — 18.57	0.003
Central isthmic PTC	4.67	1.53 — 14.21	0.007	4.55	1.46 — 14.15	0.009
Preoperative lymphadenectasis in the central zone	1.43	1.13 — 2.02	0.047			
Prelaryngeal and/or pretracheal LNM				7.39	2.84 — 23.68	0.000
Contralateral paratracheal LNM				3.14	2.46 — 8.01	0.006

LNM, lymph node metastasis; PTC, papillary thyroid carcinoma.

## Discussions

The present study suggested that young age (< 55y) and larger size (> 1cm) of the largest diameter of tumor were independent risk factors for contralateral paratracheal LNM and the presence of more than 5 metastatic lymph nodes, and that prelaryngeal and/or pretracheal LNM and contralateral paratracheal LNM were independent risk factors for the presence of more than 5 metastatic lymph nodes. Prelaryngeal and/or pretracheal LNM was established as a risk factor for contralateral paratracheal LNM among patients with lobar PTC and paracentral isthmic PTC, but it was not a risk factor for contralateral paratracheal LNM among patients with isthmic (central and paracentral) PTC. Extraglandular invasion posed a risk factor for contralateral paratracheal LNM among patients with lobar PTC and paracentral isthmic PTC. Preoperative lymphadenectasis in the central zone was a risk factor for contralateral paratracheal LNM in patients with isthmic (central and paracentral) PTC. Being located in the central isthmus was a risk factor for contralateral/bilateral paratracheal LNM and the presence of more than 5 metastatic lymph nodes in patients with isthmic PTC.

It is well known that PTC is more common in younger women (20–22). Several studies have underscored young age as a risk factor for LNM (5, 23–25), a trend also confirmed in our study. It was guessed that the carcinoma might be more aggressive in the younger patients. The size of the tumor serves as a pivotal indicator of PTC staging and exerts a significant impact on prognosis (1, 17, 26). Therefore, it holds an important position in determining the appropriate surgical approach (1, 17). The study found that greater tumor diameter heightened the likelihood of LNM, a finding consistent with prior research (5, 6, 23, 24). A larger tumor diameter signifies enhanced invasiveness and/or prolonged developmental duration, potentially elevating the propensity for LNM.

Prelaryngeal and/or pretracheal LNM and contralateral paratracheal LNM had a positive association with the presence of more than 5 metastatic lymph nodes. Central lymph node consisted of prelaryngeal lymph node, pretracheal lymph node, ipsilateral paratracheal lymph node, and contralateral paratracheal lymph node. Prelaryngeal and/or pretracheal LNM and contralateral paratracheal LNM increased the total number of LNM. Prelaryngeal and/or pretracheal LNM was a risk factor for bilateral paratracheal LNM (18, 27), therefore it might predict more metastatic lymph nodes. Contralateral paratracheal LNM might mean strong invasiveness of the tumor, which led to more metastatic lymph nodes.

Previous studies suggested that prelaryngeal and/or pretracheal LNM increased the risk of contralateral paratracheal LNM (18, 27, 28). A similar result was obtained in these patients with lobar PTC and paracentral isthmic PTC in the present study. While, this result was not found among patients with isthmic (central and paracentral) PTC. This phenomenon could be attributed to the central isthmic gland, which is directly connected to the bilateral paratracheal lymph nodes via the lymphatic vessel system, consequently facilitating bilateral paratracheal LNM. While the paracentral isthmic gland and lobar gland were indirectly connected to the contralateral paratracheal lymph nodes by the central isthmic gland and prelaryngeal and/or pretracheal lymph node. The current study has also identified being located in the central isthmus as a risk factor for contralateral/bilateral paratracheal LNM in cases of isthmic PTC, but being located in the paracentral isthmus did not significantly affect the incidence of contralateral paratracheal LNM among patients with lobar PTC and paracentral isthmic PTC compared to being located in the lobe. These observations may imply that the characteristics of paracentral isthmic PTC resemble those of lobar PTC. The present study also found that central isthmic PTC was more prone to the presence of more than 5 metastatic lymph nodes. In addition to bilateral lymphatic drainage to the bilateral paratracheal lymph nodes, the central isthmic gland is thinner compared to the paracentral and lobar glands, potentially rendering it more susceptible to extraglandular invasion and LNM. Another possible reason might be the fact that isthmic PTC was more aggressive confirmed by molecular data (7, 8).

The effect of extraglandular invasion on contralateral paratracheal LNM become significant among patients with lobar PTC and paracentral isthmic PTC. It might be because that extraglandular invasion increased the risk of prelaryngeal and/or pretracheal LNM (18, 29, 30), and then indirectly increased the risk of contralateral paratracheal LNM. Preoperative lymphadenectasis in the central zone might reflect a strong tumor aggressive, which elevated the likelihood of contralateral paratracheal LNM.

After PSM, the occurrences of inadvertent PG resection and immediate postoperative hypoparathyroidism remained elevated in the paracentral isthmic PTC cohort compared to the lobar PTC group. It might be attributed to the surgical method. Among the PSM’s patients with lobar PTC, 24, 74, and 75 patients underwent lobe thyroidectomy with ICND, TT with ICND, and TT with BCND, respectively. All the patients with paracentral isthmic PTC underwent TT with BCND.

## Conclusions

In conclusion, it was observed that a young age (<55 years) and a larger size (>1cm) of the largest tumor diameter heightened the likelihood of contralateral paratracheal LNM and the presence of more than 5 metastatic lymph nodes. Additionally, prelaryngeal and/or pretracheal LNM and contralateral paratracheal LNM were associated with an increased risk of the presence of more than 5 metastatic lymph nodes in the current investigation. Prelaryngeal and/or pretracheal LNM and extraglandular invasion increased the risk for contralateral paratracheal LNM among patients with lobar PTC and paracentral isthmic PTC. However, being located in paracentral isthmus had no significant association with contralateral paratracheal LNM and the presence of more than 5 metastatic lymph nodes in these patients. This indicated that LT with ICND might be appropriate for paracentral isthmic PTC without prelaryngeal and pretracheal LNM. Central isthmic PTC exhibited a higher propensity for bilateral paratracheal LNM and the presence of more than 5 metastatic lymph nodes in patients with isthmic PTC. Preoperative lymphadenectasis in the central zone implied an elevated likelihood of bilateral paratracheal LNM in these patients. It was implied that TT with BCND might be necessary for central isthmic PTC, especially these with preoperative lymphadenectasis in the central zone.

## Data Availability

The raw data supporting the conclusions of this article will be made available by the authors, without undue reservation.
